# Pharmacists in Federally Qualified Health Centers: Models of Care to Improve Chronic Disease

**DOI:** 10.5888/pcd16.190163

**Published:** 2019-11-21

**Authors:** Jennifer L. Rodis, Traci R. Capesius, Julie T. Rainey, Magdi H. Awad, Carrie Hornbeck Fox

**Affiliations:** 1 The Ohio State University College of Pharmacy, Columbus, Ohio; 2 Professional Data Analysts, Inc, Minneapolis, Minnesota; 3 AxessPointe Community Health Center/NEOMED, Akron, Ohio; 4 Ohio Department of Health, Columbus, Ohio

## Abstract

**Introduction:**

Pharmacists are underused in the care of chronic disease. The primary objectives of this project were to 1) describe the factors that influence initiation of and sustainability for pharmacist-provided medication therapy management (MTM) in federally qualified health centers (FQHCs), with secondary objectives to report the number of patients receiving MTM by a pharmacist who achieve 2) hemoglobin A1c (HbA_1c_) control (≤9%) and 3) blood pressure control (<140/90 mm Hg).

**Methods:**

We evaluated MTM provided by pharmacists in 10 FQHCs in Ohio through qualitative thematic analysis of semi-structured interviews with pharmacists and FQHC leadership and aggregate reporting of clinical markers.

**Results:**

Facilitators of MTM included relationship building with clinicians, staff, and patients; regular verbal or electronic communication with care team members; and alignment with quality goals. Common MTM model elements included MTM provided distinct from dispensing medications, clinician referrals, and electronic health record access. Financial compensation strategies were inadequate and varied; they included 340B revenue, incident-to billing, grants, and shared positions with academic institutions. Of 1,692 enrolled patients, 60% (n = 693 of 1,153) achieved HbA_1c_ ≤9%, and 79% (n = 758 of 959) achieved blood pressure <140/90 mm Hg.

**Conclusion:**

Through this statewide collaborative, access for patients in FQHCs to MTM by pharmacists increased. The factors we identified that facilitate MTM practice models can be used to enhance the models to achieve clinical goals. Collaboration among clinic staff and community partners can improve models of care and improve chronic disease outcomes.

**Summary**

**What is already known on this topic?**

Evidence shows that pharmacist-provided medication management can improve chronic disease outcomes; however, pharmacists are not consistently considered integral members of health care teams.

**What is added by this report?**

It provides an example of how collaboration among state public health, clinical, and academic partners can catalyze expansion of models of care that include pharmacists and that inclusion of pharmacists on care teams has the potential to improve chronic disease outcomes.

**What are the implications for public health practice?**

Findings can provide guidance to public health, clinical, and academic partners in their efforts to expand care models that include pharmacists, to help improve chronic disease outcomes.

## Introduction

Although well positioned to fill gaps in health care, pharmacists have long been underused (1,2). This is especially relevant in chronic disease management despite evidence that demonstrates pharmacists’ success in improving outcomes through collaborative care and medication therapy management (MTM) (1–6). MTM involves a multifaceted approach of reviewing medications, identifying and remedying medication-related problems, providing disease state management and self-management education, addressing medication adherence issues, and considering preventive health strategies to optimize medication-related health (3,4,7,8). An MTM service includes a comprehensive medication review to ensure that the patient’s medication-related needs have been met and all of their medications are appropriate, effective, safe, and convenient. At the end of the visit, a care plan is developed and shared with the patient and the primary care provider to resolve and prevent any drug therapy problems by eliminating unnecessary medications, initiating appropriate medications, adjusting dosage regimens, addressing adverse reactions, and increasing the patient’s willingness and ability to adhere to the medication regimen (9,10). Through MTM, pharmacists play an important role in addressing health care disparities in underserved areas (11–14). Developments including passage of the Patient Protection and Affordable Care Act (15), subsequent expansion of Medicaid, and the establishment of federally qualified health centers (FQHCs) have created more opportunities for pharmacists to provide care in community-based settings. Integration of MTM remains limited in many community-based settings due to lack of reimbursement, medical provider buy-in, time, and resources (16,17). Additionally, evidence is sparse with regard to outcomes in FQHCs and factors that facilitate initiation, continuation, and sustainability of care provided by pharmacists in FQHCs (17,18).

The Ohio Department of Health (ODH), Ohio Pharmacists Association (OPA), and Ohio Association of Community Health Centers (OACHC) collaborated with colleges of pharmacy in Ohio on a 5-year, 2-phase project to address these gaps and opportunities. This project involved developing a statewide learning community and advisory board, tracking aggregate outcomes for patients receiving care from pharmacists, and qualitatively evaluating processes surrounding pharmacist-provided MTM. The primary objectives of this project were to 1) describe factors that influence initiation of and sustainability for pharmacist-provided MTM in FQHCs, and 2) report the number of patients receiving MTM by a pharmacist who achieved hemoglobin A1c (HbA_1c_) control (≤9%) and blood pressure control (<140/90 mm Hg).

## Methods

This was a multi-site, prospective project approved by the institutional review boards of The Ohio State University and the Ohio Department of Health. A multidisciplinary consortium was created to oversee the project. The consortium set a mission to expand team-based care involving pharmacists to prevent chronic disease; disseminate outcomes to support successful models of care; and collaborate across private, public, and academic entities to promote statewide advancement in patient access to pharmacist care. Members of the consortium included representatives from the ODH, the OPA, the OACHC, all 7 colleges of pharmacy in Ohio, the state’s Medicare quality improvement organization, and pharmacists providing care to patients in FQHCs. The consortium met quarterly to guide project activities, review goals and plans for disseminating outcomes, and share updates and best practices among the FQHC pharmacists related to practice models and care strategies. The project used qualitative research methods and descriptive statistics to report on objectives.

The first project phase (Phase 1) was initiated in March 2014 and concluded in December 2016 and involved 3 FQHCs with well-established models for pharmacists to provide MTM to patients. The processes for recruitment, quantitative data reporting, and analysis for this first phase were published previously (19). Patients were recruited at each FQHC from reports created with each site’s electronic health record (EHR). Patients were included if they were aged 18 to 75 years; had a diagnosis of hypertension, diabetes, or both, with diagnosis occurring at least 1 year prior; were seen for a medical visit(s) at least once in the last year; and had a most recent HbA_1c_ >9% and/or a most recent systolic blood pressure ≥140 mm Hg or diastolic blood pressure ≥90 mm Hg. We assessed how well patients had control of their diabetes (good control, HbA_1c_ <7% to poor control, HbA_1c_ >9%) and whether patients had controlled (<140/90 mm Hg) or uncontrolled (≥140/90 mm Hg) hypertension. Visit lengths and structures varied with the pharmacist providers based on individual patient needs and clinic structures among the 10 FQHCs involved in the project. Follow-up data were gathered from EHRs in each FQHC site and reported centrally to the Ohio Department of Health for analysis. These metrics were based on Uniform Data System clinical measures defined by the Health Resources and Services Administration (HRSA) and required for reporting by FQHCs (20). Patients were excluded if they were pregnant, diagnosed with end stage renal disease, or had received a pharmacist visit at the site within 1 year before enrollment.

The second phase (Phase 2) of this project was initiated in January 2016. Investigators recruited 2 additional cohorts of pharmacists. These next 2 cohorts (4 FQHCs in the first and 3 in the final) included pharmacists with new or emerging opportunities to establish pharmacist-provided MTM in FQHCs. First and second phase sites (10 sites in total) provided MTM according to their individual clinic policies, procedures, and workflow. Inclusion and exclusion criteria as well as quantitative data reporting and analysis were the same for the first and second phases of the project (19). All 10 sites continued to enroll patients from their start date through December 31, 2017; data reporting concluded on June 30, 2018.

To understand facilitators and barriers to implementing MTM in an FQHC setting, semi-structured interviews were conducted with both clinical pharmacists and nonpharmacist clinic leaders (eg, medical directors, chief executives) recruited from FQHCs taking part in this project. The 3 sites from Phase 1 and 5 of the 7 sites from Phase 2 participated in the qualitative interviews; however, due to site staff turnover and resulting incomplete information, qualitative data from one Phase 2 site was eliminated from thematic analysis, leaving 7 total sites involved in the qualitative analysis. Comparable qualitative data were not collected from sites in the final cohort, because these sites were still in the process of initiating MTM services and could not contribute comparable data.

A single investigator identified a clinical pharmacist to be interviewed at each FQHC. Clinical pharmacists then identified nonpharmacist clinic leaders in their affiliated FQHC to be interviewed in an effort to gather more than one perspective at each FQHC and capture the nuances and complexity of MTM implementation at each site.

Interview protocols were developed by 3 investigators with input from ODH epidemiology and evaluation staff. A set of interview protocols was developed for each clinical pharmacist to capture perspectives close to the beginning of each project phase and 6 to 12 months later. A separate protocol, drawn from a subset of questions from the clinical pharmacy protocol, was developed to capture the perspectives of nonpharmacist clinic leaders. Protocols aimed to gather information about each site’s approach to implementing MTM: rationale for implementing the service; financial supports used; patient identification and referral processes; staffing; elements of the MTM model of care; and key facilitators, barriers, and lessons learned, as well as the future sustainability of MTM at each site and advice for others contemplating MTM implementation.

Two investigators conducted telephone interviews with clinical pharmacists at each of the 3 Phase 1 sites between July and August 2015 and again in January 2016. Nonpharmacist clinic leaders from these sites were interviewed in January and February 2016. A similar series of interviews was conducted with 4 Phase 2 sites by the same 2 investigators in July 2016 and again in July or August 2017. All interviewees consented to have their interviews recorded and were provided with their interview summary to review for completeness and accuracy. Corrections or additions supplied by interviewees were incorporated into the final summaries.

After finalizing all interview summaries (n = 20 interviews, n = 14 unique interviewees) across cohorts, 2 investigators conducted an inductive, cross-case thematic analysis (19) using the qualitative data analysis software NVivo11 (QSR International). Informed by analysis techniques described in Patton (21) and Charmaz (22) and to identify emergent themes, 2 investigators identified and discussed broad common themes and broke those themes down further to more nuanced themes. Throughout this process, investigators resolved any differences that arose via consensus. The themes were further vetted for cohesiveness and validity by 2 additional investigators with training and experience in clinical pharmacy and MTM. Significance throughout qualitative analysis was defined by the study team as at least 4 of the 7 sites reporting an element or theme. Quotations or excerpts from interview summaries and recordings were de-identified to protect the confidentiality of the interviewees and the FQHCs.

## Results

Seven pharmacists (2 male, 5 female) and 7 nonpharmacist clinic leaders (2 male, 5 female) from 7 FQHC sites were interviewed. Information gathered during these interviews was categorized into 3 key areas related to MTM models of care in FQHCs: common elements (Table 1), strategies for financial compensation (Table 2), and facilitators to initiation, continuation, and expansion (Table 3).

**Table 1:** Common Elements to Medication Therapy Management Models of Care in 7 Ohio Federally Qualified Health Centers (FQHCs), March 2014–June 2018

Element	7 FQHCs	4–6 FQHCs
**Clinic and pharmacy structure**		
MTM services provided onsite at FQHC	●	
Pharmacy has at least partial clinical access to EHR	●	
Collaborative Practice Agreement used		●
On-site pharmacy		●
FQHC owns pharmacy		●
**Care team members**		
Medical provider (MD, NP, PA)	●	
Pharmacist	●	
Pharmacy resident(s)		●
Pharmacy student(s)		●
**Patient identification**		
Medical provider referral	●	
Referral through EHR		●
EHR data mining		●
**Eligibility criteria**		
Uncontrolled chronic condition^a^	●	
Multiple medications (ie, polypharmacy)		●
**Visit structure and content**		
Separate visit with a pharmacist^b^	●	
MTM platform documentation and billing^c^	●	
Communication (verbal or via EHR) with clinician	●	
Medication assistance (ie, cost)		●

Abbreviations: EHR, electronic health record; MD, doctor of medicine; MTM, medication therapy management; NP, nurse practitioner; PA, physician assistant.

a Inclusion criteria required patients to have either uncontrolled hypertension (blood pressure >140/90 mm Hg) or uncontrolled type 2 diabetes (hemoglobin A_1c_ >9%).

b Two sites also conducted joint visits with a medical provider.

c Mirixa (Mirixa Corporation, Reston, Virginia) and/or OutcomesMTM (Cardinal Health, Dublin, Ohio).

**Table 2:** Medication Therapy Management (MTM) Financial Compensation Strategies Implemented in 7 Ohio Federally Qualified Health Centers, March 2014–June 2018

Site	OutcomesMTM and/or Mirixa Electronic MTM Platforms	Participation in 340B Drug Pricing Program	Medical Billing^a^	Portion of Pharmacist Salary Supported by a University	Clinic Budget or Grants	Pharmacy Budget or Grants
**1**	●	●^b^	●^c^^,^ d	●	●	
**2**	●	●^b^	●		●	
**3**	●	●^e^		●		
**4**	●	●^c^			●	
**5**	●	●^b^				
**6**	●	●^c^	●		●	
**7**	●	●^c^		●	●	●

a Billing through Evaluation and Management codes 99211–99215.

b Funds go to clinic, used to expand clinical pharmacy services, including MTM.

c Funds go to clinic, not allocated to any specific services.

d Billing through lower-level, incident-to code 99211.

e Funds go to clinic, used to support patient care generally. No information available about allocation of funds to MTM or other clinical pharmacy services.

**Table 3:** Facilitators to Initiation, Continuation, and Expansion of Medication Therapy Management Models of Care in 7 Ohio Federally Qualified Health Centers, March 2014–June 2018

Theme (No. of Sites Contributing to Theme)	Selected Representative Statements
**Facilitators to Initiation**	
Identify or cultivate a champion in administration, quality improvement committee, or C-suite (n = 7)	The administrative team and the board of directors were all supportive of MTM from the beginning. The CEO is a registered nurse with a strong clinical background and understood the need for MTM.
	The CMO has a history of working with clinical pharmacists for most of her career. One of the primary preceptors (a physician) had a BS in pharmacy as an undergraduate. The CEO of the clinic is also supportive of pharmacy being an integral part of the clinic. The support is embedded within the culture. The clinic is extremely supportive of pharmacy.
Engage clinician champions (n = 7)	The associate medical director indicated relying on pharmacists to help provide education and follow-up support to her patients. This carries over into new clinician orientation where she talks about how helpful support from pharmacists has been to her and her patients and encourages them to take advantage of on-site MTM services.
	The clinical pharmacist reports that open communication with clinicians and finding clinician champions early on who are supportive of a pharmacist’s role on the care team are important. Champions can be used as a sounding board and can relay to other clinicians how pharmacists can complement their work with patients.
	At first the clinical pharmacist worked exclusively with one NP who had some previous experience working with a pharmacist. This NP became a champion and served as a model for other clinicians. The NP would identify 10 to 20 of his patients with the greatest needs who had upcoming appointments and ask the clinical pharmacist to work with them. Through this collaboration, they were able to capture data to show the benefit of MTM.
Ensure pharmacists have support to conduct MTM outside of medication dispensing (n = 7)	The CMO remarked that it is often difficult for a dispensing pharmacist to have time to conduct MTM. Having a clinical pharmacist and resident, and sometimes students, who can conduct or help with MTM has been key.
	The clinical pharmacists work alongside the medical providers and not in the dispensary.
Align the potential benefits of MTM with FQHC quality care goals (patient experience, health outcomes, clinical quality measures) (n = 7)	From the start of MTM, administrators were excited about MTM because of the potential it held for improving patient outcomes
	Reimbursement was not as important to administrators as improving quality of patient care and, along with that, quality measures.
	MTM improves the quality of patient care . . . and helps them achieve their goals as a patient-centered medical home.
Educate clinicians on how pharmacists can contribute to the care team (n = 4)	In the beginning, to help foster buy-in among clinicians, the clinical pharmacist held monthly 1-hour meetings to present the project and to describe how the pharmacist planned to communicate with the clinicians about patient care.
	Before implementation of MTM, the clinical pharmacist attended medical staff meetings. She introduced the program in advance so that everyone was clear about what it offered and worked to establish relationships with clinicians in advance.
	The CEO noted that initially some clinicians and staff had a tough time grasping the idea of having a pharmacist on the care team, so the clinical pharmacist started out providing some basic information to clinicians such as what MTM is and how to use pharmacy services.
**Facilitators to continuation and expansion**	
Collect data on patient outcomes/quality of care; share with clinicians and management (n = 7)	Collecting, tracking, and sharing outcome data with clinicians and management were very important. The clinical pharmacist had a plan from the beginning as to how they were going to use the data to increase buy-in and support for MTM. They track 3 types of data: physician perspectives, patient perspectives, and patient outcomes, for example, hemoglobin A1C, blood pressure, and LDL cholesterol. Without this evidence they would not have support continued for their efforts.
	The CEO noted that once a practice is able to document positive patient outcomes and share those outcomes with clinicians, they see the value of it. The clinical pharmacist produces a quarterly newsletter that includes a patient story. The CEO finds this has been an effective communication strategy for clinicians and staff.
Show how clinical pharmacy services benefit the care team (n = 7)	The associate medical director noted that having pharmacists on the care team really enhances the team: “[The pharmacist's efforts] could serve as a text-book example of what team-based care looks like in a PCMH.”
	The CEO remarked that physicians support MTM because the program allows them to do their job. They do not have extensive time to speak with patients about medication adherence or to provide the lengthy conversations needed to help patients who are confused, elderly, cannot read, or just cannot understand. Clinicians know if they hand these patients off to the pharmacist that it makes their day go more smoothly.
Seek and illuminate the financial benefits of MTM to the clinic (n = 7)	The executive director and chief financial officer have always been supportive of pharmacy services, but as reimbursement is starting to be tied to it (eg, quality of care, reduced hospital readmissions), there is a greater focus on this type of service.
	They also plan to continue having conversations with third-party payers (eg, MCOs) around direct reimbursement for MTM.
	Clinic management and physicians see the benefit of investing 340B revenue into clinical pharmacy services because it improves patient outcomes.
	There was no expectation from the FQHC that MTM should generate revenue to support the clinical pharmacist’s salary. But as the project developed, he began to plan for ways to make MTM sustainable post grant. He wanted to be able to show the project’s worth, and also to avoid having the position be a cost burden.
Communicate regularly with clinicians (in person or via EHR) (n = 6)	The clinic workstation is shared by all of the clinicians, and the clinical pharmacist finds this helps facilitate collaboration across staff and clinicians.
	All pharmacists are also invited to attend the monthly clinician meeting. In the past, these meetings were only for physicians and nurse practitioners. The pharmacists requested to be invited to attend those meetings as well. This allows pharmacists a chance to interact with clinicians outside of the clinic and the opportunity to hear what they are hearing from administration.
	Now that the clinical pharmacist has access to the health center's EHR, they can document visit notes and recommendations directly into the EHR as they meet with patients.
Show how MTM contributes to meeting clinic goals (n = 6)	The associate medical director finds that providing MTM makes it easier for the clinic to reach its quality goals and make improvements in quality measures, for example hemoglobin A1c levels for diabetes.
	Focusing on quality measures was already a priority at this organization, so the MTM team worked to incorporate improvement in these measures as a priority.
	External factors such as quality measures certainly influence clinicians’ and administration’s willingness to take on MTM. The clinical pharmacist expects they will have the data they need to demonstrate these improvements to providers and administration.
Build relationships with clinicians (n = 5)	Where the clinical pharmacist sees the greatest need for clinical pharmacy is in support of midlevel clinicians (eg, nurse practitioners and physician assistants) and is working on building relationships with these clinicians.
	Getting buy-in can be a challenge but is critical. The clinical pharmacist suggests that pharmacists work alongside physicians as much as possible, spend time at the nurses’ station, stay in communication, and get to know the medical assistants. Other care team members don’t necessarily know what pharmacists can do, so they need to be there to show them what they can do. It is important to build these relationships and know that this might take time.
	There is still more work to be done, however, to build support for MTM among clinicians. Some clinicians still don’t trust the service, or they just get caught in their old routines and don’t think about how the pharmacist can help them. The clinical pharmacist thinks that continuing to build relationships with each clinician, by helping answer their patient’s questions, will help her build buy-in for working with a larger number of patients in a more in-depth manner.

Abbreviations: 340B, 340B drug pricing program; BS, bachelor of science; C-suite, top senior staff within an FQHC; CEO, chief executive officer; CMO, chief medical officer; EHR, electronic health record; FQHC, federally qualified health center; LDL, low-density lipoprotein; MCOs, Medicaid-managed care organizations; MTM, medication therapy management; NP, nurse practitioner; PCMH, patient-centered medical home.

Elements of clinic structure, workflow, and patient care processes common to all sites providing MTM in FQHCs were pharmacists providing MTM services billed through Medicare Part D–integrated platforms (eg, Mirixa [Outcomes Incorporated], OutcomesMTM [Cardinal Health]), at least partial pharmacist access to EHRs, a care team minimally inclusive of a medical provider and a pharmacist, and referral of patients to pharmacists by a medical provider. With regard to clinic operations, a notable commonality described among all MTM models was patient visits with pharmacists separate from dispensing functions (Table 1).

Strategies for financial compensation for MTM also demonstrated commonalities with all sites engaging in billing through MTM platforms and every FQHC reporting involvement in a 340B drug pricing program. Other strategies for financial compensation were mixed and included clinic or pharmacy grants, collaboration or shared funding with a college of pharmacy, and billing with evaluation and management medical codes either as incident-to or through shared medical visits (Table 2).

Facilitators were organized based on whether they were related to MTM initiation or continuation and expansion (Table 3). In addition to the top facilitators described in Table 3, the need for sustainable compensation for pharmacists providing MTM emerged as another significant theme among pharmacists and nonpharmacist leaders. The lack of adequate levels and modalities of reimbursement for care was described as a major barrier to initiation and expansion of MTM. Interviews across all sites mentioned the importance of recognition of pharmacists as providers and the need for appropriate financial compensation for care provided by pharmacists. For example, one clinic leader shared:

Recognizing pharmacists as providers at the federal level would help with reimbursement for services. If the provider status of pharmacists is ever approved, they will be able to obtain adequate reimbursement for Medicaid and Medicare, can bill under the pharmacists’ name, and will be properly paid for their time and effort. Depending on the degree to which this actually happens and how many insurance providers will accept the change in status, [pharmacists] could add more clinical positions and not rely solely on medication dispensing. If this were approved, [pharmacies] would hire more pharmacists, serve many more patients, and could provide clinical services at locations where they don’t have a dispensing component.

Other themes emerged but did not reach significance, including workflow and clinical infrastructure considerations and staff education. More specifically, a few sites mentioned the importance of clinicians directly referring patients to pharmacists, availability of private rooms for pharmacist and patient meetings, clinicians’ prior experience collaborating with pharmacists on patient care, and pharmacists with past experience providing comprehensive MTM as being important facilitators of MTM initiation. Educating nonclinicians and other clinic staff on MTM (what it entails, benefits of) was mentioned by a few sites as important to obtaining buy-in and support for MTM, along with educating patients and clinicians to improve understanding and participation.

**Figure 1:**
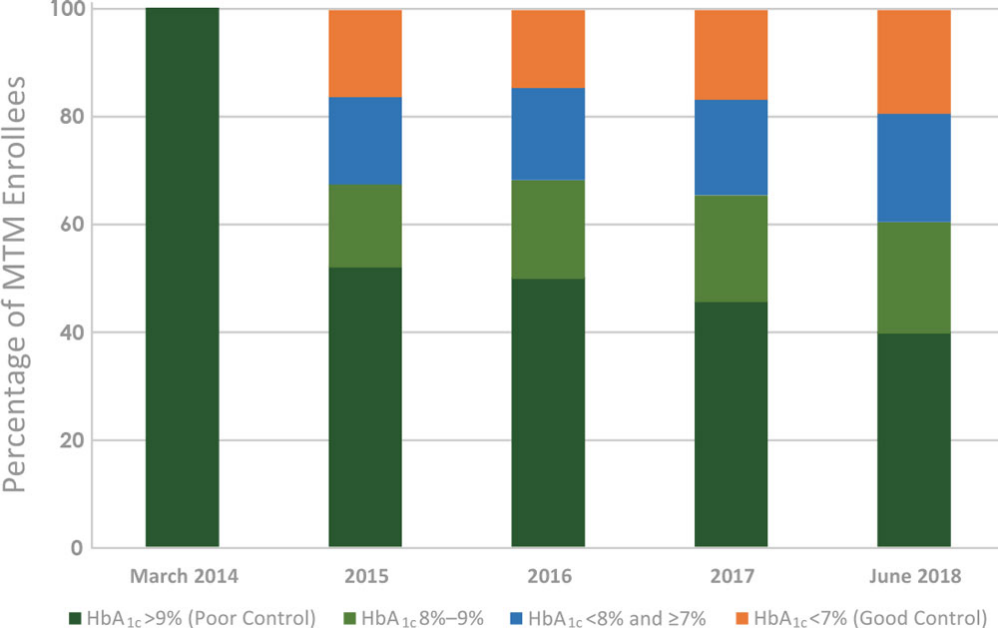
Aggregate achievement of HbA_1c_ goals of patients enrolled in medication therapy management (MTM) services at 10 Ohio federally qualified health centers from March 2014 through June 2018. Abbreviation: HbA_1c_, hemoglobin A1c.

**Figure 2:**
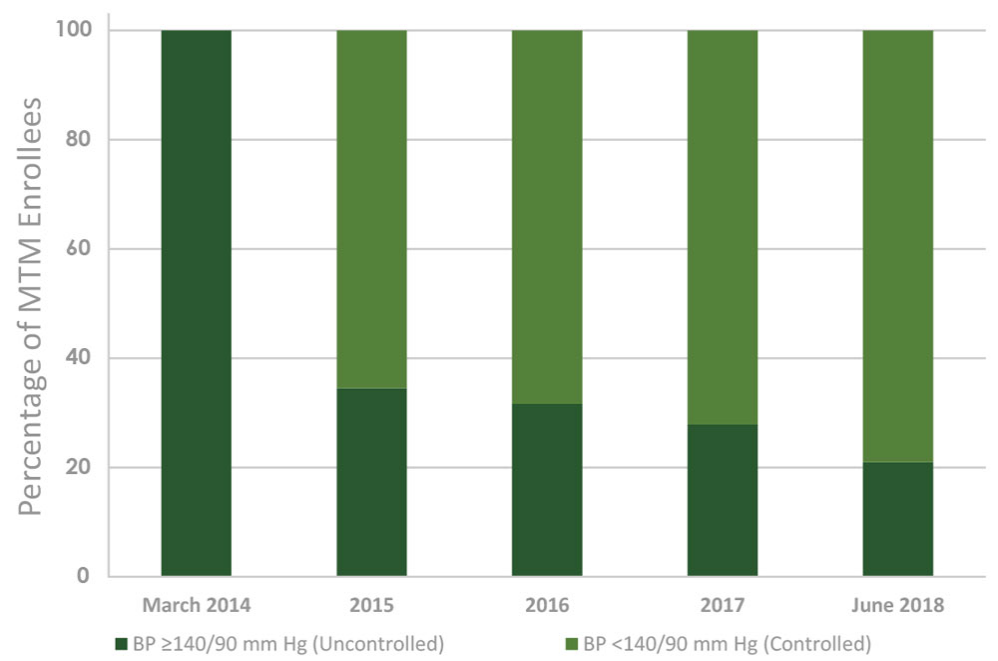
Aggregate achievement of blood pressure (BP) goals of patients enrolled in medication therapy management services at 10 Ohio federally qualified health centers from March 2014 through June 2018.

Between March 2014 and December 2017, 1,692 patients were enrolled in this study at the 10 FQHCs in all phases of the project; 1,153 of these patients were enrolled with uncontrolled diabetes, and 959 of these patients were enrolled with uncontrolled hypertension. At final data collection ending on June 30, 2018, approximately 60% (n = 693) of patients with uncontrolled diabetes achieved an HbA_1c_ ≤9%, 20.6% (n = 238) between 8% and 9%, 20.2% (n = 233) between 7% and <8%, and 19.3% (n = 222) <7% (Figure 1). Of those with hypertension, 79% (n = 758) achieved a blood pressure that was in range at <140/90 mm Hg (Figure 2).

**Table:** 

Date	HbA_1c_ >9% (Poor Control)	HbA_1c_ 8%–9%	HbA_1c_ <8% and ≥7%	HbA_1c_ <7% (Good Control)
	Percentage of MTM Enrollees			
**March 2014**	100	0	0	0
**2015**	52.2	15.5	16.2	16.2
**2016**	50.1	18.3	17.2	14.4
**2017**	45.8	19.8	17.8	16.6
**June 2018**	39.9	20.6	20.2	19.3

**Table:** 

Date	BP ≥140/90 mm Hg (Uncontrolled)	BP <140/90 mm Hg (Controlled)
	Percentage of MTM Enrollees	
**March 2014**	100	0
**2015**	34.6	65.5
**2016**	31.6	68.4
**2017**	27.8	72.2
**June 2018**	21.0	79.0

## Discussion

Semi-structured interviews with pharmacists and FQHC leadership identified common elements of MTM workflow among sites and key facilitators to initiation, continuation, and expansion of these services. Patients with previously uncontrolled diabetes and blood pressure displayed aggregate achievement of HbA_1c_ and blood pressure goals following visits with pharmacists.

The degree of clinical goal achievement in this project was comparable to results reported by the Patient Safety and Clinical Pharmacy Services Collaborative (PSPC), a national initiative designed by HRSA in 2008 to enhance medication use in safety-net organizations, including FQHCs. In 2012, PSPC reported achievement of goals, with 35% of PSPC sites attaining desired HbA_1c_ levels and 43% of PSPC sites reporting meeting hypertension goals. In the Ohio project, 60.1% of patients achieved HbA_1c_ goals and 79.0% reported achievement of hypertension targets (23). The Change Package initiative with PSPC provided implementation steps and best practice tips from FQHCs with established pharmacy services. The Change Package recommendations align with themes that emerged in the Ohio MTM analysis. Similar facilitators between the 2 included identifying physician champions, providing EHR access for pharmacists, sharing outcomes from pharmacy services with clinic leadership and clinicians, educating clinicians on benefits of clinical pharmacy services, and pharmacists engaging in regular communication with clinicians and care team members (18).

Investigations have demonstrated strategies to build successful pharmacist-provided MTM in community-based settings, such as FQHCs. Pestka et al (17) proposed a stepwise process for community pharmacies to integrate MTM into practice sites. With the focus on traditional community pharmacies, their findings were aimed mainly at the internal pharmacy staff and considerations for changes within the pharmacies. In our qualitative analysis of pharmacists in FQHCs, it is notable that many of the key facilitators to initiation involved stakeholders external to the pharmacists and pharmacy staff, such as clinicians, patients, and clinic leadership. Snyder et al (16) evaluated 3 community-based models of care including an independent pharmacy, a chain pharmacy, and an FQHC practice model. Barriers to MTM in these settings included reimbursement as well as lack of provider buy-in, time, resources, and collaborative practice agreements (CPAs). Facilitators included team-based care and collaboration with academic partners. Jorgensen et al (24) conducted telephone interviews with pharmacists, physicians, and nurse practitioners from 23 health care teams that had integrated a new pharmacist role and identified 7 key themes describing the barriers and facilitators the teams experienced during pharmacist integration. The themes identified in their study aligned and reinforced results described in this project, including the importance of relationship-building, experience of providers working with pharmacists, and the need for adequate resources and funding. Finally, Fischer et al (25) conducted a mixed-methods cohort study in one FQHC with a pharmacy to examine the implementation and impact of a broad program involving MTM. Interviews identified enabling factors to success that align with our results, including data access, leadership support, staffing, and 340B funding. 

Our findings correlate well with other pharmacist service–specific literature, which confirms and expands the evidence base for pharmacist-provided care in FQHCs. However, no previous study involved the breadth and number of FQHCs and interview participants as the Ohio project. Additionally, no previous study described a state-specific learning community. The state-focused collaboration involving the OPA, all 7 colleges within the state, the ODH, and OACHC facilitated a learning and practice advancement consortium with shared payor opportunities as well as pharmacy practice act considerations.

Strategies for financial compensation varied among the sites involved in this statewide project. Sites identified that improvement in compensation opportunities for pharmacists as providers of care is needed and may be necessary for continued expansion of pharmacy services in FQHCs. Murawski et al (26) evaluated practice characteristics and reimbursement for pharmacists in certified collaborative clinical practice in New Mexico and North Carolina and found, as we did, that despite integration and acceptance of pharmacists providing care by patients and clinicians, reimbursement challenges continued to limit expansion of the model.

### Limitations

Individual pharmacists and FQHCs developed the workflow models that fit with their infrastructure, resources, and patient populations. Thus, these individual processes of care may have influenced results by introducing unknown confounders because quantitative data was not analyzed at the individual FQHC site or patient level. Patient experience with the pharmacist-provided care was not evaluated in this study and is an opportunity for future evaluation. Transcripts from interviews conducted with first phase, experienced sites as well as second phase, emerging sites were compiled and analyzed as one group of data. Data from pharmacist and nonpharmacist interviewees were also analyzed in aggregate and included 7 of the 10 pharmacy sites. An additional confounder was that a few questions were developed and added mid-study, based on information volunteered by some early interviewees. Themes that arose from responses to those questions were less likely to reach thematic significance. For example, expansive CPA legislation involving pharmacists and physicians were passed in Ohio in 2016 while this project was in process. A consistent theme or commonality in CPAs may have arisen if all interviewees had been asked to discuss CPAs during all interview phases.

### Conclusion

Statewide collaboration among state public health, FQHCs, pharmacists, and colleges can catalyze expansion of pharmacist models of care and improve chronic disease outcomes. Through this statewide collaborative, patients cared for in FQHCs had access to pharmacist-provided MTM services for diabetes and/or blood pressure management. Although this statewide public health collaboration model with pharmacy is transferable to other states, key elements to patient care models and facilitators to success that were identified can be applied at the clinic site level to build successful MTM models of care in FQHCs. Pharmacists and other health care providers and policy makers must continue to strive for sustainable financial compensation to improve patient access to pharmacist-provided MTM.
